# Objective Estimates of Physical Activity and Sedentary Time among Young Adults

**DOI:** 10.1155/2017/9257564

**Published:** 2017-01-02

**Authors:** Jessica L. Unick, Wei Lang, Deborah F. Tate, Dale S. Bond, Mark A. Espeland, Rena R. Wing

**Affiliations:** ^1^Brown University and The Miriam Hospital's Weight Control and Diabetes Research Center, Providence, RI, USA; ^2^Wake Forest School of Medicine, Winston-Salem, NC, USA; ^3^Gillings School of Public Health, University of North Carolina, Chapel Hill, NC, USA

## Abstract

*Background*. This study examines factors associated with physical activity (PA) and sedentary behaviors (SB) in young adults (18–35 years) and compares objective and subjective assessment measures of PA and SB.* Methods*. 595 young adults (27.7 ± 4.4 years; 25.5 ± 2.6 kg/m^2^) enrolled in the Study of Novel Approaches to Weight Gain Prevention (SNAP) trial. Hours/day spent in SB (<1.5 METs) and minutes/week spent in bout-related moderate-to-vigorous intensity PA (MVPA; ≥3 METs and ≥10 min) were assessed using self-report and objective measures. Demographic factors associated with SB and MVPA were also explored (i.e., age, gender, BMI, ethnicity, work and relationship status, and number of children).* Results*. Objective MVPA (263 ± 246 min/wk) was greater than self-report estimates (208 ± 198 min/wk; *p* < 0.001) and differed by 156 ± 198 min/wk at the individual level (i.e., the absolute difference). Females, overweight participants, African Americans, and those with children participated in the least amount of MVPA. Objective estimates of SB (9.1 ± 1.8 hr/day; 64.5% of wear time) were lower than subjective estimates (10.1 ± 3.5 hr/day; *p* < 0.001), differing by 2.6 ± 2.5 hr/day for each participant.* Conclusion*. Young adults interested in weight gain prevention engage in both high levels of MVPA and SB, with participants self-reporting fewer MVPA minutes and more SB compared to objective estimates. This study is registered at ClinicalTrials.gov (NCT01183689).

## 1. Introduction

Both physical inactivity and excessive sedentary time are independent risk factors for the development of cardiovascular disease [1–3]. Further, physical activity (PA), mainly that which is performed in structured bouts and at higher intensities, plays a significant role in weight control, particularly in the prevention of weight gain or weight regain following weight loss [4]. However, despite these known health benefits of PA and reduced sedentary time, it is concerning that individuals spend an average of 8 hours/day engaging in sedentary behaviors [5] and a large percentage of the population does not engage in sufficient amounts of PA [6]. One particular segment of the population that is at high risk for low PA and high sedentary time are young adults.

Young adulthood, or the period of time between 18 and 35 years of age, is considered to be a highly transitional period, with many young adults experiencing significant life changes associated with pursuing further education, building a career, getting married, or having children. Perhaps due to these competing life demands, young adulthood is also characterized by significant weight gain [7, 8] and reduced PA [6, 9]. Cross-sectional studies indicate that a significant proportion of young adults participate in insufficient levels of moderate and vigorous intensity PA [6, 9, 10]. Moreover, a reduction in PA throughout young adulthood has also been observed in longitudinal cohorts [11, 12], with greater reductions in PA observed among those getting married, becoming a parent, or moving into a “live-in” relationship [11], suggesting that PA may vary across different segments of the young adult population.

To date, the majority of research examining PA and sedentary time in young adults has utilized self-report measures. This is of concern given that self-reported PA is often prone to response bias (e.g., social desirability and inaccurate recall), and thus it is unclear whether the relationships between PA and sedentary time with weight as well as other health parameters remain when PA is assessed objectively. However, of the few studies which have utilized objective measures, only 10.8% of young adults aged 20–29 years engaged in an “adequate” amount of MVPA according to guidelines [6]. Moreover, objectively assessed sedentary time has been shown to increase within young adulthood [13] and a sample of overweight/obese young adults were found to spend 65% of their waking hours engaging in sedentary behaviors [13].

Overall, few studies have assessed PA and sedentary time in young adults, particularly using objective assessment measures. Moreover, little is known about the extent to which demographic factors (e.g., age, gender, and BMI) or life stage (e.g., married, student) influence PA or sedentary behaviors in this population. A better understanding of these factors could have important implications for clinicians or researchers promoting PA or weight control. Finally, it is unclear how objective and subjective measures of PA and sedentary time compare to one another in this population. The concordance between these types of measurement methods is critical for informing future PA and obesity-related research studies and allowing investigators and clinicians to more accurately compare findings between studies which utilize self-report versus objective measures. Such a comparison may be particularly important given that there is no true “gold standard” for assessing PA in a free-living environment and also that objective PA monitoring may not be feasible to implement within all studies due to budgetary constraints, the number of participants enrolled, or participant burden. It is for these reasons that it is critical to examine not only PA and sedentary behaviors in young adults but also predictive factors of PA and sedentary time, using both subjective and objective measures.

The purpose of this study is to objectively quantify PA and sedentary time across the entire spectrum of young adulthood (age 18–35 years) and to examine the percentage of individuals who meet the national PA guidelines within a unique subgroup of the young adult population, those interested in weight gain prevention. Further, we examine whether time spent in sedentary behaviors and bout-related MVPA differs by age, gender, BMI, relationship, employment, and parental status. Finally, a secondary aim is to examine the concordance between objective and self-report measures of MVPA and sedentary time, as this will be useful when interpreting and comparing data across studies which utilize dissimilar PA assessment methodologies.

## 2. Methods

### 2.1. Participants and Procedures

Participants were individuals enrolled in the Study of Novel Approaches to Weight Gain Prevention (SNAP) trial. The SNAP trial is a randomized clinical trial examining two novel approaches to weight gain prevention in young adults (“Large Changes” and “Small Changes” conditions) compared to a minimal treatment control group (“Self-Guided” condition). Participants randomized to the “Small Changes” group were instructed to reduce their dietary intake by 100 kcals/day and increase their PA by adding 2000 steps/day. “Large Change” participants were instructed to lose 5–10 lbs within the first 4 months to create a buffer against future weight gain and engage in ≥250 min/week of MVPA. Full inclusion and exclusion criteria for the SNAP trial have been previously published [14, 15]. In short, SNAP participants were aged 18–35, had a BMI between 21 and 30 kg/m^2^, and were excluded if they reported a recent weight loss >10 pounds, had a current or recent pregnancy, or had any other health condition that could affect the safety of PA or weight loss. The current analyses focus on the baseline assessment period.

All measures reported in this paper were collected prior to randomization, at one of two screening visits. At screening visit 1, height and weight were objectively measured and participants were fitted with a SenseWear Armband (SWA) and instructed to wear this device during all waking hours (except while bathing or swimming) for seven consecutive days. Between screening visit 1 and screening visit 2, participants completed a series of questionnaires online via a secure server, which included a demographics questionnaire as well as the Sedentary Behavior Questionnaire (SBQ; see description below). At screening visit 2 (approximately 1 week after screening visit 1), participants completed a modified version of the Paffenbarger Physical Activity Questionnaire (PPAQ; see description below), which was administered by interview. Baseline assessments for the SNAP trial were conducted between November 2010 and February 2012. All study procedures were approved by The Miriam Hospital's (Providence, RI) and the University of North Carolina's (Chapel Hill, NC) Institutional Review Boards.

### 2.2. Measurement of Physical Activity and Sedentary Time

#### 2.2.1. SenseWear Armband (SWA)

The SWA (BodyMedia, Pittsburgh, PA) was used to objectively assess PA and sedentary time. Participants were instructed to wear the SWA during waking hours, except while bathing or swimming. This monitor is worn on the back of the upper arm and assesses PA using a biaxial accelerometer and a unique combination of heat sensors (heat flux, galvanic skin response, skin temperature, and near body temperature). Data collected through these sensors are integrated via proprietary algorithms to provide minute-by-minute estimates of energy expenditure, assigning a metabolic equivalent (MET) value to each minute the monitor is worn. Energy expenditure estimates produced by the SWA have previously been shown to be valid when compared to indirect calorimetry [16] and doubly labeled water [17]. Further, group-level SWA estimates have been found to be similar to other hip worn accelerometers [18].

The SenseWear Professional software (version 7.0) was used to calculate SWA wear time and minute-by-minute MET values. From these data, time spent in sedentary activities (<1.5 METs) and light (1.5 to <3.0 METs), moderate (3.0 to <6.0 METs), vigorous (≥6 METs), and moderate-to-vigorous intensity PA (MVPA; ≥3.0 METs) was determined and expressed as total daily time (e.g., min/week or hours/day) as well as the percentage of total wear time spent at a particular intensity. In addition, bout-related MVPA was calculated by summing the number of minutes spent in any activities that were ≥3.0 METs and ≥10 minutes in duration, allowing for a 1-minute interruption in MVPA. Only participants meeting valid wear time requirements (≥4 days and ≥8 hours/day) were included in the analyses. Further, only days in which the monitor was worn for ≥8 hours were used.

#### 2.2.2. Paffenbarger Physical Activity Questionnaire (PPAQ)

A modified version of the PPAQ was used to capture self-reported PA over the previous 7 days and is the same version of the questionnaire that was implemented within the Early Adult Reduction of weight through Lifestyle intervention (EARLY) trials consortium, which included the SNAP trial. This interviewer-administered questionnaire queried participants on (1) the number of flights of stairs climbed each day, (2) time spent brisk walking in bouts ≥10 minutes in duration (this includes walking outside, at an indoor facility, or on a treadmill), and (3) time spent engaging in sports and recreational activities (excluding occupational or job related activities or activities <10 minutes in duration) over the previous 7 days. A modified version of the PPAQ was used in order to assist participants with estimating their walking minutes and to capture more purposeful activity (which is similar to the bout-related MVPA derived from the SWA).

The modified PPAQ differed from the original PPAQ in several ways. First, unlike the original PPAQ which asks participants to report on the number of city blocks walked each day, the modified version asked participants to report on the number of days/week and minutes/day spent brisk walking, since the number of city blocks walked is often difficult for participants to estimate. Second, to capture more purposeful activity, participants were instructed to only include brisk walking which was ≥10 minutes in duration. These 10-minute criteria were a modification from the original PPAQ. Further, sports/recreational activities that were <10 minutes or <3 METs were not included in the analyses. Finally, the original PPAQ provides estimates of PA expressed as kcals/week and sums the energy expenditure of stair climbing, walking, and sports/recreational activities. In the current study, the outcome measure was minutes/week spent in bout-related MVPA (minutes/week was not converted to kcals/week) and did not include stair climbing, since it is unlikely that participants climbed stairs for ≥10 minutes at a time. Only sports/recreational activities that were at least a moderate intensity (i.e., ≥3 METs) according to the Compendium of Physical Activity [19] were included in the this weekly PA total. Thus, both the SWA and PPAQ provided estimates of bout-related MVPA (≥3 METs and ≥10 minutes in duration), expressed in minutes/week.

#### 2.2.3. Sedentary Behavior Questionnaire (SBQ)

The SBQ used in the EARLY trials was a modified version of the SBQ developed for the Coronary Artery Risk Development in Young Adults (CARDIA) study (http://www.cardia.dopm.uab.edu/images/more/pdf/year25/cardia/form91.pdf). Participants were asked to report on the amount of time that they spent engaging in eight types of sedentary behaviors on a typical weekday or weekend day. The 6 questions original to the CARDIA study asked about non-work-related sedentary time while the two additional questions added by the EARLY Consortium investigators queried participants on the amount of time that they spent sitting at work or school doing computer and noncomputer work. Participant responses (response categories were none, 15 minutes or less, 30 minutes, 1, 2, 3, 4, 5, or 6 hours, or more) were summed and truncated at 24 hours if the sum was >24. A weighted average of weekday and weekend day sedentary time was calculated to determine the mean daily time (hours/day) spent in sedentary behaviors across an entire week.

### 2.3. Statistical Analyses

Descriptive statistics included mean and standard deviation (SD) or median and interquartile range (IQR) for continuous measures, depending on the normality of distribution, and count and percentage for categorical variables. Differences in bout-related MVPA and sedentary time among demographic groups, such as gender, age, BMI, race, marital status, number of children at home, being a full time worker, and being a full time student, were evaluated using the Wilcoxon rank sum test for two group comparisons and the Kruskal-Wallis test for three or more group comparisons. Adjusted means, standard errors, and adjusted *p* values were obtained from analysis of covariance models, which included clinic, age group, race, marital status, children in household, full time work, and daily wear time. For the dichotomous outcome of ≥150 minutes/week of bout-related MVPA, the Chi-Square test or Fisher's exact test were used to obtain unadjusted *p* values for group differences. To obtain adjusted *p* value, we fit the generalized linear model with logit link function, adjusting for clinic, age group, race, marital status, children in household, full time work, and daily wear time. In order to compare PA levels between SWA and PPAQ, SWA data were standardized to a 1-week period by multiplying average daily MVPA minutes by 7. Both SWA and PPAQ estimates of bout-related MVPA were used to determine whether individuals fell above or below the national PA guideline of ≥150 minutes/week of MVPA. Analyses were performed using the SAS version 9.4 (SAS Institute, Cary, NC).

## 3. Results

### 3.1. Participants

A total of 599 participants enrolled in the SNAP trial, 99% (*n* = 595) of whom met the minimal wear time criteria at baseline (≥4 days of ≥8 hours of wear time per day) and thus were included in the analyses. Participants were predominately white (72.9%), 27.7 ± 4.4 years of age, and 78.2% were female. The mean BMI was 25.5 ± 5.6 kg/m^2^ and 45.9% of participants were of normal weight and the remaining 54.1% were overweight. On average, participants wore the SWA for 7.1 ± 0.8 days for 14.1 ± 1.5 hours/day. Participants without complete data on the SBQ (*n* = 33) were excluded from all analyses which utilized this questionnaire.

### 3.2. Objectively Assessed Sedentary and Physical Activity Behaviors

Time spent in sedentary, light, moderate, and vigorous intensity activities, as measured objectively by the SWA, is shown in Table 1. On average, participants spent 263.0 ± 246.4 min/week in bout-related MVPA and 60.2% met the national PA guideline of ≥150 minutes/week of bout-related MVPA. After adjusting for SWA wear time and other demographic variables, females, overweight participants (i.e., BMI 25–30 kg/m^2^), African Americans, and those with children participated in fewer minutes/week of bout-related MVPA compared to males, normal weight participants, Caucasians, and those with no children (Table 2). Fewer overweight participants, African Americans, and individuals with children met the ≥150 min/week threshold for bout-related MVPA compared to those who were of normal weight, Caucasian, or did not have any children. There was also a trend for fewer females to achieve this MVPA threshold compared to males.

On average, participants spent 9.1 ± 1.8 hours/day (64.5% of total wear time) engaging in sedentary behaviors as measured objectively. Overweight participants and those without children engaged in the greatest amount of sedentary time.

### 3.3. Level of Agreement between Objective and Questionnaire Measures

#### 3.3.1. Moderate-to-Vigorous Intensity PA

There was a significant correlation between objective (i.e., SWA) and subjective (i.e., PPAQ) measures of bout-related MVPA (*r* = 0.56, *p* < 0.001). However, examination of group-level means revealed that bout-related MVPA was 55.2 ± 242.9 minutes/week higher when assessed by SWA, compared to PPAQ (Table 3). Further, this discrepancy was greater when examined at the individual level, with an average difference between SWA and PPAQ for each participant of 156.2 ± 194.0 min/week. This discrepancy was the largest in normal weight males (251.9 ± 293.9 min/wk) and the smallest in overweight females (129.4 ± 185.9). Finally, a greater number of participants met the MVPA threshold of ≥150 minutes/week when assessed by SWA, compared to PPAQ (60.2% versus 54.1%).

#### 3.3.2. Sedentary Time

There was a modest yet significant correlation between SWA and SBQ-assessed hours/day spent in sedentary behaviors (*r* = 0.27, *p* < 0.001). At the group level, participants reported engaging in 0.9 ± 3.5 hours/day more in sedentary behaviors compared to when objectively measured (Table 4). Interestingly, this group-level discrepancy between SWA and SBQ was only observed in females, and not males. At the individual level, the average difference between SWA and SBQ was 2.6 ± 2.5 hours/day and this discrepancy was >2 hours/day in both males and females.

## 4. Discussion

The SNAP trial examines PA and sedentary behaviors among normal weight and overweight, nonobese, young adults who have an interest in weight gain prevention. Overall, study participants engaged in approximately 260 min/week of objectively assessed bout-related MVPA and nearly two-thirds of these young adults achieved or exceeded the national PA guidelines for improved health (≥150 min/week) according to objective measures. While this amount of PA is much higher than what has been reported in the general population, of concern is the fact that nearly two-thirds of the time that the PA monitor was worn, participants were engaging in sedentary behaviors. This pattern of high MVPA coupled with high sedentary time has been referred to as the “active couch potato” phenomenon and carries important health risks given that excessive sedentary time is associated with poorer cardiometabolic health, independent of MVPA [20].

This was one of the few studies to date to examine objective MVPA among young adults. Using the same PA monitor, Jakicic et al. [21] previously reported that young adults with a BMI of 25–40 kg/m^2^ (70% female, mean age = 30.9 years) were engaging in 430 MET-min/week, which is equivalent to approximately 100–125 min/week of bout-related MVPA. Further, NHANES participants between the ages of 20–29 years averaged 80 min/week of MVPA (assessed via the Actigraph) which is considerably less than the 263 min/week observed in the current study [6]. While it is not entirely clear why young adults in the current study had much higher PA levels than what has been previously reported, we hypothesize this may be in part due to the fact that participants in the current study were interested in weight gain prevention and thus may have been more health conscious and aware of the importance of regular PA. Further, differences in the objective PA monitor employed (e.g., SWA versus Actigraph) [6] or the body weight of study participants (e.g., normal weight/overweight versus obese) [21] may have led to these discrepant findings.

From a clinical perspective, it is also important to identify factors associated with MVPA in young adults. In the current study, participants who were overweight, female, and African American or had children were least likely to engage in higher levels of MVPA. However, unlike previous self-report PA studies which suggest that PA is reduced throughout young adulthood [11, 12], our data do not support the hypothesis that age is a driving factor for PA. While there was a trend for MVPA to decrease by approximately 20 minutes/week every 5 years in the current study, this trend disappeared after adjusting for other demographic factors. Further analyses reveal an interaction between age and number of children, and thus it is likely that children not age may lead to reductions in PA with increasing age.

It is clear that participants in the current study engaged in high levels of PA; however, they also engaged in a large amount of sedentary time (9.1 hours/day or 64.5% of total armband wear time). This magnitude of sedentary time is much greater than the 7.5 hours/day (54% of total wear time) which was objectively measured in NHANES young adults [22]. It is possible that this discrepancy could be attributed to the differences in the study samples, the measurement device, or the location of the PA monitor (upper arm versus waist-mounted). However, in a sample of overweight/obese young adults, participants engaged in nearly an identical amount of sedentary time as observed in the current study, despite engaging in significantly less MVPA compared to our SNAP participants [13]. It is plausible that SNAP participants, although more health conscious, may be less aware of the negative effects of sedentary time as they are aware of the positive effects of regular PA. Given the current findings, additional intervention efforts may be needed for reducing sedentary time, in addition to increasing or maintaining PA levels, among young adults interested in weight gain prevention.

A secondary aim of this study was to compare subjective and objective measures of PA and sedentary time. While previous research suggests that individuals tend to self-report greater PA compared to objective estimates [23, 24], findings from the current study indicate the reverse, with participants engaging in an additional 55 minutes/week of objectively assessed MVPA compared to self-report estimates. Further, within an individual, the absolute difference between measures was very high (156 min/wk) and this discrepancy varied by gender and weight category with SWA-derived MVPA estimates being greater than PPAQ estimates in normal weight men and women and normal weight women, but not overweight women. There are many possible explanations for the discrepancy between SWA and PPAQ. First, MVPA was assessed using an absolute cut-point (i.e., 3 METs) versus a relative intensity cut-point (e.g., ≥ 55% of maximal heart rate); thus, it is possible that ambulating at 3-4 METs did not “feel like” moderate-intensity PA for many SNAP participants, and therefore they did not report it as such. Second, the PPAQ assesses leisure-time activities such as sports and recreational activities or walking done for the purpose of exercise or transportation. It does not include job related or household activities; thus, it is possible that if a participant engaged in household or occupational activities of ≥3 METs sustained for ≥10 minutes, these would have been captured by the SWA and not the PPAQ. The current findings suggest that, for PA and obesity clinicians and researchers, it may not be appropriate to compare PA estimates derived from studies which rely on self-report versus objective PA assessment techniques. Moreover, when discussing PA with their patients, clinicians may want to consider that individuals in this age group may overreport SB and underreport PA compared to objective monitors.

Subjective and objective estimates of sedentary time were also compared and participants self-reported engaging in an additional hour per day of sedentary time compared to when measured objectively, although this discrepancy was only seen in females and was independent of BMI. The correlation observed between SBQ and SWA for sedentary time was modest (*r* = 0.27) and was similar to that reported by Barone Gibbs et al. [13] in an overweight/obese young adult cohort. These data suggest that self-report and objective measures of sedentary time may not be interchangeable. Further, a limitation of the SWA is that it is unable to differentiate sitting from standing and thus future studies should use objective PA devices with an inclinometer to verify these findings.

This study is important because it objectively assessed PA and sedentary behaviors in young adults, an underresearched subgroup of the adult population that is at risk for weight gain and reductions in PA. Further, it is strengthened by a large sample size, the use of self-report and objective PA, and sedentary measures, and it provides additional information about activity patterns among individuals interested in weight gain prevention. However, it is not without limitations. First, it is unknown whether wearing the armband device for 7 days could have influenced activity patterns. Also, the PPAQ and SBQ are self-report measures of PA with inherent limitations. Further, the week that participants may have used to report their PA on the PPAQ may not have exactly matched up with the same week that the armband was worn. Similarly, the SBQ asks participants to report how many minutes or hours/day spent engaging in different types of sedentary behaviors; however, the response options (none, <15 min, 30 min, 1 hour, 2 hours, 3 hours, 4 hours, 5 hours, and 6+ hours) force participants to estimate sedentary time so that it conforms to one of the response categories. Moreover, participants were instructed to report their sedentary behaviors for a typical weekend or weekday, while it is unknown whether the armband was worn during a “typical” week or how many weekdays and weekend days that the armband was worn. Finally, approximately three-quarters of the study participants were female or white and baseline PA levels were high; thus, it is unclear whether these findings would generalize to other young adult samples.

In conclusion, this study adds to the scant literature which objectively assesses PA and sedentary time in young adults. Further, it suggests that young adults who enroll in a weight gain prevention treatment program are highly active, despite still engaging in moderately high levels of sedentary time. Women, African Americans, and those who are overweight or have children are most likely to engage in the least amount of PA; thus, intervention efforts may prioritize on these subgroups. Finally, this study suggests that much more research is needed to fully understand the activity patterns of young adults, particularly given the limited concordance between self-report and objective measures of PA and sedentary time.

## Figures and Tables

**Table 1:** Objectively assessed time spent in physical activity and sedentary behaviors among 595 participants enrolled in the SNAP trial.

	Total time (mean ± SD)	Total time median (IQR)	% of daily wear time (mean ± SD)	% of daily wear time median (IQR)
Sedentary (min/wk)	3828.3 ± 757.0	3833.2 (3350, 4284)	64.5% ± 9.9	65.3% (57.9%, 71.6%)
Light (min/wk)	1477.4 ± 452.8	1429.0 (1119, 1781)	25.1% ± 7.6	24.5% (19.3%, 30.3%)
Moderate (min/wk)	580.0 ± 309.2	513.8 (359.0, 720.0)	9.8% ± 5.1	8.6% (6.3%, 12.2%)
Vigorous (min/wk)	38.8 ± 59.6	16.0 (2.8, 46.4)	0.64% ± 0.96	0.27% (0.05%, 0.76%)
MVPA (min/wk)	618.8 ± 335.1	543.0 (375.0, 779.6)	10.4% ± 5.5	9.1% (6.5%, 13.0%)
Bout-related MVPA (min/wk)	263.3 ± 246.3	358.0 (86.0, 358.0)	4.4% ± 4.1	3.3% (1.5%, 6.0%)

MVPA: moderate-to-vigorous PA mean ± SD; median (interquartile range).

**Table 2:** Objectively assessed physical activity and sedentary time stratified by demographic characteristics among SNAP study participants.

	*N* (%)	Bout-related MVPA (min/week)				% of individuals with ≥150 min/wk of bout-related MVPA			Sedentary time (hours/day)			
		Mean ± SD	*p* value	Adjusted means ± SE	Adjusted *p* value^*∗*^	*N* (%)	*p* value	Adjusted *p* value^*∗*^	Mean ± SD	*p* value	Adjusted mean ± SE^*∗*^	Adjusted *p* value^*∗*^
*Overall*	595	263.3 ± 246.3				358 (60.2%)			9.1 ± 1.8			
*Gender*			<0.001		<0.001		0.03	0.07		<0.001		0.07
Males	130	335.2 ± 294.0^a^		330.7 ± 34.2^a^		89 (68.5%)			9.6 ± 1.8^a^		9.0 ± 0.2	
Females	465	243.2 ± 227.6^b^		226.8 ± 27.5^b^		269 (57.9%)			9.0 ± 1.8^b^		8.8 ± 0.2	
*Age (years)*			0.07		0.96		0.03	0.77		0.15		0.55
18–24	168	281.9 ± 234.8		275.2 ± 34.6		113 (67.3%)			9.1 ± 1.7		8.8 ± 0.2	
25–29	204	265.7 ± 247.6		278.1 ± 32.9		124 (60.8%)			9.3 ± 1.7		8.9 ± 0.2	
30–35	223	247.2 ± 253.6		283.0 ± 29.0		121 (54.3%)			9.0 ± 2.0		9.0 ± 0.2	
*BMI (kg/m* ^*2*^)			<0.001		<0.001		<0.001	0.002		0.95		0.01
21–25	273	327.9 ± 284.8^a^		341.3 ± 30.6^a^		187 (68.5%)^a^			9.1 ± 1.7		8.7 ± 0.2^a^	
25–30	322	208.6 ± 192.4^b^		216.2 ± 30.0^b^		171 (53.1%)^b^			9.1 ± 1.9		9.0 ± 0.2^b^	
*Ethnicity*			<0.001		0.015		0.001	0.01		0.04		0.10
AA	66	164.5 ± 160.0^a^		226.6 ± 38.6^a^		26 (39.4%)^a^			9.2 ± 1.4^a,b^		9.0 ± 0.2	
White	434	274.5 ± 248.0^b^		316.9 ± 27.5^b^		272 (62.7%)^b^			9.0 ± 1.8^a^		8.7 ± 0.2	
Other	95	281.1 ± 273.2^a,b^		292.8 ± 35.4^a,b^		60 (63.2%)^b^			9.6 ± 2.0^b^		9.0 ± 0.2	
*Full time work*			0.19		0.11					0.97		0.68
Yes	374	251.9 ± 234.7							9.1 ± 1.7		8.9 ± 0.2	
No	221	282.6 ± 264.3							9.1 ± 2.0		8.9 ± 0.2	
*Relationship*			0.04		0.06		0.13	0.47		0.01		0.52
Single	178	298.2 ± 250.1^a^		265.5 ± 27.0		118 (66.3%)			9.5 ± 2.2^a^		9.0 ± 0.2	
In relationship	221	252.9 ± 235.8^a,b^		221.5 ± 25.9		133 (60.2%)			9.0 ± 1.6^b^		8.9 ± 0.2	
Married	188	235.7 ± 232.5^b^		233.4 ± 23.8		102 (54.3%)			8.9 ± 1.5^b^		9.1 ± 0.1	
Separated^*ǂ*^	8	425.9 ± 539.3^a,b^		394.7 ± 84.4		5 (62.5%)			7.7 ± 2.4^a,b^		8.6 ± 0.5	
*Children in household*			<0.001		0.03		<0.001	0.001		<0.001		<0.001
No children	475	282.7 ± 251.7^a^		331.2 ± 25.8^a^		309 (65.1%)^a^			9.3 ± 1.8^a^		9.3 ± 0.2^a^	
1 child	55	186.5 ± 249.4^b^		259.8 ± 39.7^b^		19 (34.6%)^b^			8.7 ± 1.8^a^		9.0 ± 0.2^a^	
2+ children	57	178.0 ± 156.7^b^		245.3 ± 41.9^b^		24 (42.1%)^b^			8.2 ± 1.6^b^		8.3 ± 0.2^b^	

Unadjusted means ± SD; MVPA: moderate-to-vigorous intensity physical activity; AA: African Americans; ^*ǂ*^ “Separated” category includes those who reported being separated, widowed, or divorced; *∗* indicates adjusted model which includes armband wear time and all other demographics factors identified in this table. Groups (e.g., males and females) within the same category (e.g., gender) that have dissimilar superscripts indicate that the categories are significantly different from one another (*p* < 0.05).

**Table 3:** Self-report (PPAQ) versus objective (SWA) assessment of moderate-to-vigorous intensity physical activity among SNAP participants.

	*N* (%)	SWA Bout-related MVPA (min/wk)	PPAQ Bout-related MVPA (min/wk)	Difference in weekly bout-related MVPA (SWA − PPAQ)	*p* value SWA versus SBQ	Absolute value of bout-related MVPA (min/wk)	SWA % achieving ≥ 150 min/wk	PPAQ % achieving ≥ 150 min/wk	% achieving ≥ 150 min/wk: *p* value SWA versus PPAQ
*Total sample*	595	263.3 ± 246.3 185.5 (85.8, 358.0)	208.2 ± 197.9 165.0 (60.0, 295.0)	55.2 ± 242.9 31.4 (−47.0, 141.0)	<0.001	156.2 ± 194.0 93.0 (39.0, 209.0)	358 (60.2%)	322 (54.1%)	0.04
NW & F	235	307.4 ± 260.0 248.0 (116.0, 423.5)	218.2 ± 192.6 162.0 (76.0, 315.0)	89.1 ± 222.9^a^ 59.0 (−23.2, 185.0)	<0.001	162.1 ± 176.8^a,b^ 106.6 (45.0, 210.4)	158 (67.2%)	128 (54.5%)	0.005
OW & F	230	177.7 ± 165.3 143.5 (50.8, 253.8)	199.2 ± 209.6 163.5 (60.0, 269.0)	−21.5 ± 225.7^b^ −1.3 (−82.3, 57.0)	0.26	129.4 ± 185.9^a^ 68.1 (32.1, 170.0)	111 (48.3%)	123 (53.5%)	0.26
NW & F	38	454.9 ± 386.5 349.7 (178.0, 612.0)	251.1 ± 225.0 225.0 (100.0, 345.0)	203.8 ± 330.0^a^ 161.5 (0.0, 340.0)	<0.001	251.9 ± 293.9^c^ 211.0 (66.0, 340.0)	29 (76.3%)	26 (68.4%)	0.44
OW & M	92	285.7 ± 230.9 216.5 (123.5, 413.7)	187.2 ± 165.9 140.0 (50.0, 265.0)	98.6 ± 236.8^a^ 74.2 (−26.2, 185.5)	<0.001	168.1 ± 193.2^a,b,c^ 98.5 (45.4, 240.5)	60 (65.2%)	45 (48.9%)	0.03

NW: normal weight; OW: overweight; F: female, M: male; data are presented as mean ± SD followed by median (interquartile range); weekly bout-related MVPA: minutes/week spent engaging in moderate-to-vigorous intensity physical activity ≥10 minutes in duration; SWA: SenseWear armband; PPAQ: Paffenbarger Physical Activity Questionnaire. % Within a column, means sharing the same superscript are not significantly different from each other, after adjusting for clinic, age, race, marital status, children in household, full time student/working status, and daily wear time (*p* < 0.05).

**Table 4:** Self-report (SBQ) versus objective (SWA) assessment of sedentary behaviors among participants enrolled in the SNAP trial.

	*N* (%)	SWA sedentary time (hours/day)	SBQ sedentary time (hours/day)	Difference in sedentary time hours/day (SWA − SBQ)	*p* value for difference between SWA and SBQ	Absolute difference in sedentary time (Hours/day)^*∗*^
*Total sample*	562	9.1 ± 1.8 9.1 (8.0, 10.2)	10.1 ± 3.5 9.6 (7.9, 11.9)	−0.9 ± 3.5 −0.5 (−2.6, 1.2)	<0.0001	2.6 ± 2.5 1.8 (0.8, 3.4)
Normal weight, female	225	9.1 ± 1.8 9.1 (8.0, 10.3)	9.9 ± 3.1 9.7 (7.9, 11.7)	−0.9 ± 2.9 −0.4 (−2.7, 1.1)	0.0002	2.3 ± 2.0 1.9 (0.8, 3.0)
Overweight, female	215	8.9 ± 1.8 8.8 (7.7, 10.0)	10.2 ± 4.0 9.8 (7.9, 12.4)	−1.4 ± 4.0 −0.7 (−3.0, 1.2)	<0.0001	3.0 ± 3.0 1.9 (1.1, 3.9)
Normal weight, male	37	9.5 ± 2.4 9.4 (8.3, 10.2)	9.4 ± 2.9 8.9 (7.4, 11.0)	0.0 ± 2.9 0.1 (−1.8, 1.3)	0.7505	2.1 ± 1.9 1.7 (0.9, 2.6)
Overweight, male	85	9.7 ± 1.5 9.5 (8.7, 10.5)	10.2 ± 3.2 9.4 (8.5, 11.4)	−0.5 ± 3.6 −0.3 (−1.3, 1.7)	0.7528	2.5 ± 2.6 1.7 (0.7, 3.4)

Mean ± SD, median (interquartile range); SWA: SenseWear armband; SBQ: Sedentary Behavior Questionnaire. Absolute difference of the two measures (computed by taking the *absolute value* of the discrepancy between the self-report and objective measures for each individual, and then taking the average of these individual differences). The difference and absolute difference in sedentary time was not significantly different across the 4 groups (*p* ≥ 0.05).
